# From scientific article to press release to media coverage: advocating alcohol abstinence and democratising risk in a story about alcohol and pregnancy

**DOI:** 10.1080/13698575.2016.1229758

**Published:** 2016-09-20

**Authors:** Ellie Lee, Robbie M. Sutton, Bonny L. Hartley

**Affiliations:** ^a^ School of Social Policy, Sociology and Social Research, University of Kent, Canterbury, UK; ^b^ School of Psychology, University of Kent, Canterbury, UK; ^c^ Department of Psychology, Social Work and Counselling, University of Greenwich, London, UK

**Keywords:** risk, alcohol, pregnancy, democratisation, IQ, uncertainty

## Abstract

In this article, we follow the approach taken by Riesch and Spiegalhalter in “Careless pork costs lives’: Risk stories from science to press release to media’ published in this journal, and offer an assessment of one example of a ‘risk story’. Using content and thematic qualitative analysis, we consider how the findings of an article ‘Fetal Alcohol Exposure and IQ at Age 8: Evidence from a Population-Based Birth-Cohort Study’ were framed in the article itself, the associated press release, and the subsequent extensive media coverage. We contextualise this consideration of a risk story by discussing a body of work that critically engages with the development and global proliferation of efforts to advocate for alcohol abstinence to pregnant (and pre-pregnant) women. This work considers the ‘democratisation’ of risk, a term used to draw attention to the expansion of the definition of the problem of drinking in pregnancy to include any drinking and all women. We show here how this risk story contributed a new dimension to the democratisation of risk through claims that were made about uncertainty and certainty. A central argument we make concerns the contribution of the researchers themselves (not just lobby groups or journalists) to this outcome. We conclude that the democratisation of risk was advanced in this case not simply through journalists exaggerating and misrepresenting research findings, but that communication to the press and the initial interpretation of findings played their part. We suggest that this risk story raises concerns about the accuracy of reporting of research findings, and about the communication of unwarrantedly worrying messages to pregnant women about drinking alcohol.

## Introduction

In an article published previously in this journal, Riesch and Spiegelhalter discuss what they call ‘risk stories’. ‘Health related risk stories are a particular staple in press reporting on emerging science’, they note, and it is often the case that such stories concern ‘lifestyle’ focusing on ‘how common habits (usually involving food or drink) can either enhance or reduce various risks’ (2011, p. 48). We discuss here one example of a health-related risk story, in order to further engage and discuss points made in their analysis.

Our example is reporting of research about drinking alcohol during pregnancy and IQ levels in children. The research in question was published in November 2012 in the journal *PLOS One* as an article: ‘Fetal Alcohol Exposure and IQ at Age 8: Evidence from a Population-Based Birth-Cohort Study’ (Lewis et al., 2012). In the article, the authors attempted to take a novel approach to studying the relationship between foetal alcohol exposure and children’s subsequent development, by considering the effects of variations in genes that appear to be associated with the metabolisation of alcohol. In the media coverage of the article, it was argued that the findings meant there was now new, compelling scientific evidence that made strong warnings about the dangers of any drinking in pregnancy warranted. As a journalist writing in *The Times* put it, when discussing the research and its implications:

…scientists were able to see through a statistical fog to conclude that most children have genes that make it risky for their mothers to drink even a small amount in pregnancy. The results should prompt women to think again about having any alcohol at all during pregnancy, experts said. (Smyth, 2012)

The research made a big media impact, and in what follows we present a detailed analysis of how the risk of any drinking in pregnancy was represented in the media. In particular, we highlight claims that were made about the relation between the findings of research and certainty about the harm caused to child development by drinking in pregnancy (a relation *The Times* called, in the above extract, seeing through ‘a statistical fog’). Our aim, however, is not simply to provide an analysis of media framing of risk. Rather, following Riesch and Spiegelhalter (2011), we present an account of how this risk story developed and detail the relation between the research article, press communications, and media coverage. Our concern, therefore, is not only with the media coverage and its messages, but it is also with the relation between the media coverage and research as reported in a scientific article itself.

The case study discussed here has a specific context, including in health policy, out of which the claims made about risk, evidence, and uncertainty emerged. This is what has been termed the ‘medicalisation’ and ‘democratisation’ of the problem of drinking alcohol in pregnancy (Armstrong, 2003; Armstrong & Abel, 2000). We now briefly discuss this context to further situate our study. In particular, we draw attention to the question of uncertainty and evidence, as a part of this context.

## Drinking in pregnancy: the medicalisation and democratisation of risk, and the question of uncertainty

Mothers’ drinking has been a long-standing focus for campaigns seeking to highlight the dangers of alcohol. As studies of the history of these campaigns have shown, the definition of this problem that emerged from the 1970s onwards is different to that which animated concerns at other historical moments. It is different, for example to that associated with the early twentieth century temperance movement, and it is also different to that which emphasises the specific problem of female alcoholism and argues that this should be addressed by health-care systems (Golden, 2005).

Present debates about drinking and pregnancy arise out of a context in which concerns about alcohol generally have been described as medicalised; the social problem of alcohol has been increasingly linked to the development of specific diseases and disorders, physical and mental, and ill health more widely (Armstrong, 2003). For pregnancy, the medicalisation of drinking has particular features. Most notably, it has become strongly foetus focused. Claims about the problem have focussed, for example, not on the woman and the problem of drinking for her health and life as a mother, but rather on the way alcohol impairs foetal development and leads to disability and ill health in children born to mothers who drink. Alcohol abstinence during (and before) pregnancy is now advocated, and officially promoted in many countries’ public health policies on this basis. A body of work published from the late 1990s onwards has provided a critical engagement with this development (Armstrong, 1998, 2003; Bell, McNaughton, & Salmon, 2009; Golden, 1999, 2005; Lee, 2014; Leppo, Hecksher, & Tryggvesson, 2014; Lowe & Lee, 2010; Lupton, 2012, 2014; Murphy, Sutton, Douglas, & McClellan, 2011; Potter, 2012; Ruhl, 1999; Sutton, Douglas, & McClellan, 2011).

A further theme in this work is what Armstrong and Abel (2000) term the democratisation of risk and Fetal Alcohol Syndrome (FAS) is at the centre of the argument about how this framing of risk has developed (Armstrong, 1998, 2003). FAS is a serious medical condition, identifiable by a cluster of specific symptoms in babies (including retarded growth pre- and/or postnatally, abnormalities of the face including a flattened nose, very rounded eyes, and heavy, drooping eyelids, and intellectual impairment, and developmental delay). First identified in the 1970s in the United States, FAS, it was initially argued, was associated with very heavy drinking in pregnancy and identified in babies born to a very specific subgroup of the female population – alcoholic women, usually those also in poverty (Armstrong & Abel, 1999). However, in common with other social problems which may be deemed initially to effect only minorities of the population, arguments came to be pressed which emphasised the ‘universalism’ of harm caused by drinking in pregnancy. The definition of the problem of drinking in pregnancy developed rapidly in the United States through the 1980s in such a way as to present the problem as large in scale, and so overcome perceived difficulties of focusing on certain population groups, for example those in poverty or from particular ethnic minorities.

This ‘democratisation’ process was initially confined to the United States. Those advocating for the need for greater recognition of the problem of drinking in pregnancy successfully emphasised both how reported cases occurred in a wide variety of ethnic groups and social strata, and that there was just one common, causal factor: alcohol consumption. Thus, it came to be argued that ‘every woman was equally at risk’ (although evidence showed that not even every chronic alcoholic woman gave birth to an affected child, and that other factors, most notably poverty leading to poor diet, were significant) (Armstrong, 1998, p. 2028). Testament to the success of this advocacy was the development of the first abstinence advice issued in 1981 in the United States; when the then US Surgeon General advised, ‘women who are pregnant (or considering pregnancy) not to drink alcoholic beverages and to be aware of the alcoholic content of foods and drugs’ (Armstrong, 2003, p. 90). FAS, in this way, became in the United States an ‘equal opportunity disorder’ (Armstrong & Abel, 2000, p. 279). Subsequently, a range of new terms emerged to name the problem said to occur in much larger numbers of children than those previously diagnosed; these included ‘fetal alcohol effects’, ‘alcohol-related birth defects’, ‘possible FAS’, ‘partial FAS’, ‘alcohol-related neurodevelopmental disorder’ and that now used most widely, ‘Fetal Alcohol Spectrum Disorder’ (Armstrong, 1998, 2003; Lee, 2014).

Research this century has considered how this democratised account of risk to the health of the foetus/baby has diffused to and developed in many countries. It has explored features of associated policy and legal regimes that advise women not to drink and considered the perceptions and experiences of people in a context where alcohol abstinence in pregnancy is widely advised, including pregnant women, and relevant medical professionals (Armstrong, 2003; Hammer & Inglin, 2014; Holland, McCallum, & Walton, 2016).

One aspect of democratisation that emerges as important throughout all of this work is the question of uncertainty; that is, on what basis is it argued that any drinking of any amount of alcohol in pregnancy is dangerous? What is the relation between evidence and ‘no drinking’ advice? For example, as a recent research about the experiences of women in Australia reported in this journal found, ‘negotiating uncertainty’ dominates women’s experience of pregnancy (Holland et al., 2016). One reason why uncertainty emerges so manifestly in this way is because the expansion of abstinence advocacy has occurred alongside continuing considerable ambiguity in the research literature. Systematic reviews have failed, for example, to identify a relation between ‘low’ and ‘moderate’ drinking and aspects of children’s impaired development (Henderson, Kesmodel, & Gray, 2007). Further, several studies have observed a *positive* relationship between ‘low or moderate’ drinking during pregnancy and children’s subsequent developmental outcomes (Alati et al., 2008; Humphriss, Hall, May, Zuccolo, & Macleod, 2013; Kelly et al., 2012; O’Callaghan, O’Callaghan, Najman, Williams, & Bor, 2007). The ascent of abstinence advocacy has, therefore, proceeded against a backdrop of evidence that contradicts it.

The literature, because of this, has considered how policy messages are accounted for and justified. In particular, it has highlighted the rise of a precautionary approach that circumvents the uncertainty that arises from evidence. For the United Kingdom, for example, advice changed to advocate ‘avoid alcohol’ in 2007, and the most recent advice similarly advises women not to drink at all in pregnancy (and also when planning a pregnancy) (DH, 2016). This policy has emerged, however, on the basis of acknowledged uncertainty about the detrimental effects of drinking in pregnancy for child development (Lowe & Lee, 2010). In this framework, a democratised account of risk relies not on the claim that there is evidence that any drinking is harmful and so all babies are at risk, but rather that there is an absence of evidence of safety, and it is this which means no woman should drink at all. In relation to this context, the case study considered here is particularly interesting because, as we will show, strong claims were pressed that ‘new research’ now provided ‘new certainty’ and ‘settled confusion’. Indeed, this theme was central to the risk story and, it can be argued, added a novel and important dimension to the democratisation of risk, as we now discuss.

## Methodology

Riesch and Spiegelhalter’s work sought to ‘carefully examine’ risk stories by considering in detail ‘how the language and topics develop from the actual science involved, through the press release, and eventually to news coverage’ (2011, p. 48). Our approach similarly was to provide a critical and comparative examination of reportage in different stages of public communication in this case; our study therefore comprised analysis of the components that together made up the risk story. We used publicly available documents only, because the intention was to assess public communication about new research looking at drinking and pregnancy (the study therefore raised no particular ethical considerations). The components of the story were the research article itself (Lewis et al., 2012), a press release and other materials used for media communications, and the media coverage.

Items were initially accessed via the *PLOS One* website. As well as the article, we accessed the press release about the study (produced by the University of Bristol); media communications documents published by the Science Media Centre (SMC) (a London-based organisation that describes itself as ‘an independent press office helping to ensure that the public have access to the best scientific evidence and expertise through the news media when science hits the headlines’ [SMC, 2016]); and, a catalogue of media coverage stored on the website. A search of LexisNexis for the time period 15 November 2012 (the date of the press release) to 13 November 2015 was also carried out using the terms ‘pregnancy, ‘alcohol’, and ‘IQ’ to check for further coverage. In total, 65 items of media coverage were identified for analysis. The large majority of the media coverage was news reporting. The study was reported on in broadsheet and tabloid newspapers published in the United Kingdom and also in some international papers (published in the United States and Ireland) (*n* = 23). It was also reported about in online pieces for websites of UK broadcast media (such as Channel 4 News) and international equivalents (for example Fox News) (*n* = 6), and there was extensive coverage in online media of a variety of specialist and non-specialist sorts (including postings on sites hosted by RTT News, US News; *Nursing Times, Health Canal*; Wellcome Trust) (*n* = 31). Separate to news reporting, commentary about the study was not extensive. Comment pieces were published in one national newspaper, on the NHS Choices (a National Health Service website), and on four blogs (see Appendix 1).

The analysis of these documents comprised two phases, which used standard methods. Phase One aimed to generate an overall picture of the content of the three document types. On the basis of reading and rereading, all of the documents to be analysed, we identified seven main themes. (These appear in Table 1, numbered 1–7.) We then analysed the documents to identify themes present in each individually, and each document was coded as 1 or 0 according to whether the theme was present or absent. Given the purpose of the study was to compare across the document types; we than compared the occurrence of these themes between the document types. (In addition to these central themes, we identified sub-themes within each category. Due to considerations of space, we summarise these in Appendix 2.) Phase Two was a thematic qualitative analysis, which looked in more depth at each of these themes, paying attention to how they were discussed in all of the documents individually. In regards to media coverage, particular attention was paid to repetition of phrases, the origins of repeated phrases (e.g., did these phrases come from the press release), the use of expert comment, and the presence of counterclaims or alternative interpretations within each theme.

**Table 1.** Occurrence of themes in documents.

	Lewis et al.’s (2012) article	Press release	Science Media Centre ***(12 pieces)***	Media coverage ***(65 articles)***
Theme code and description	*Theme present? 1 = yes, 0 = no*		*Number of articles where theme was present*	
**1. Description of findings** (statements about the relation between IQ and drinking in pregnancy identified by the research – specifically, that children of mothers who drink have higher/lower IQs)*	1/0	0/0	1/0	7/14
**2. Interpretation of findings** (attribution of deleterious causal link between drinking and IQ)	0	1	1	53
**3. Warnings to women** (advice not to drink in pregnancy)	0	1	6	47
**4. Role of genes in moderating effects of alcohol** (the independent effect of genes in influencing alcohol metabolism)	1	1	4	55
**5. Association between IQ, drinking in pregnancy and socio-economic status** (factor that confounds associations between drinking itself and IQ)	1	0	1	5
**6. Research limitations** (reasons why the methodology or findings of the research are provisional)	1	0	6	24
**7. Addressing uncertainty in other research** (reasons why the study addresses questions other research is not able to consider)	1	1	1	30

*The figure to the left of the slash in each cell refers to correct descriptions of the observed positive correlation between drinking during pregnancy and children’s IQ. The figure to the right of the slash refers to incorrect descriptions of this relationship (i.e., statements that it was negative).

## Findings

The outcomes of the Phase One coding are set out in Table 1.

We now turn to discuss these findings, together with those of the qualitative analysis. We discuss each component of the risk story in turn (the article, then the communications to the media, then the media coverage) and draw attention to how the story developed from the original article itself, through to the media coverage.

### The original article

As we have noted, in ‘Fetal Alcohol Exposure and IQ at Age 8: Evidence from a Population-Based Birth-Cohort Study’ (Lewis et al., 2012), the authors suggest that their aim was to take a novel approach to studying the relationship between fetal alcohol exposure and children’s subsequent development. Their starting point was the question of uncertainty. More particularly, it was a form of uncertainty given by the fact that studies of the relationship between prenatal alcohol exposure and children’s later development have been hampered by a serious problem. Specifically, mothers’ alcohol intake and their socio-economic status (SES) are confounded: more high SES women report drinking during pregnancy, more low SES women report abstaining. Since higher SES is associated with better developmental outcomes including intelligence, it is difficult to separate its influence from that of alcohol consumption. In fact, several studies, *including* Lewis et al.’s (2012) own discussed here, have found that children of mothers who drink moderately during pregnancy do significantly better than those of mothers who abstain altogether. When SES is controlled for, the apparent benefit of alcohol consumption is nullified (although seldom reversed).

Instead of attempting to statistically control for SES, Lewis et al. (2012) examined the effects of variations in genes that appear to be associated with the metabolisation of alcohol. These mutations, unlike mothers’ alcohol consumption, are not confounded with SES. Lewis et al. (2012) made two crucial assumptions about these genetic variations: they assumed that they are responsible for slow or ineffective metabolisation of alcohol, and that foetuses with these genetic mutations, or whose mothers have them, thus experience greater exposure to alcohol in the womb. Of course, this second assumption can only be true of mothers who do not abstain from alcohol. So, the authors were particularly interested in the interaction between these genes and mothers’ alcohol intake (dichotomised as either light-to-moderate drinking or abstinence). Analysing data from a population-based study of over 4000 children and their mothers, they found that a set of four genetic mutations were associated with lower IQ at age 8, but only among children of mothers who had not abstained from alcohol whilst pregnant.

When we applied our coding scheme to the article by Lewis et al. (2012) and looked closely at how the seven themes identified were discussed, we found the following. Five themes were present in the article. First, given the focus of the article, Theme 4 (the role of genes in moderating effects of alcohol) was of course present. Second, the article stated accurately that the children of abstinent mothers turned out to be *less*, rather than more intelligent, than the children of mothers who drank during pregnancy (our Theme 1, ‘description of findings’). ‘For all categories of allele score [that is, the measures used of variations in genes], drinking during pregnancy was associated with a *higher* IQ score in the child’, state the authors (p. 4, our emphasis). In line with other research that, as noted above, found the same, the authors ascribe this finding to confounding factors surrounding SES (our Theme 5). They explain:

Moderate drinking in our study was found to be strongly associated with an increase in maternal age, increase in maternal education level and a higher social class all of which are associated with a higher IQ among children. Thus observed associations [that correlate drinking and higher child IQ] are probably due to confounding by socio-economically clustered factors. (p. 7)

Third, the authors acknowledge ‘research limitations’ (our Theme 6); they noted, of their findings about differences in IQ, that, ‘the effects of genotype appear modest’ (p. 7). Theme 7, ‘addressing uncertainty in other research’ was also present in the paper, however. The authors suggest that in contrast with other studies, because of its focus on genes, their research provides relative certainty, since, ‘associations between genetic variants and disease are not generally susceptible to confounding by lifestyle factors’ (p. 1). Our analysis also revealed the following about this theme. While the authors report associations between drinking and SES factors (Theme 5), they do not cite any test of whether the relationship between drinking and SES disappears, or reverses, when the SES confounds are controlled for. Neither did they report any test of whether the IQ advantage of children of drinking (vs. abstinent) mothers is statistically significant. Thus, no firm evidential basis was provided for the claim that associations are due to confounding.

Two themes were absent from the paper. The theme that pregnant women should abstain from drinking (Theme 3) did not appear at all. Neither did Theme 2 appear (attribution of causal link between drinking and IQ). However, there was ambiguity about this theme. On one hand, the authors adopt the perfectly appropriate conclusion that the gene–environment interactions they observed, ‘can be taken as providing evidence supporting a causal association with the outcome, although causation cannot be proven by the current study design’. On the other, they refer to the gene–environment interaction as if it were clearly a measure of ‘fetal exposure to alcohol’, which intuitively would be associated in readers’ minds as maternal intake of alcohol. This happens at the very beginning of the paper, in its title, ‘Fetal alcohol exposure and IQ at age 8’, which makes no mention of genes or the gene–environment interaction, and it also happens at the conclusion of the paper, where the authors write that their study, ‘offers some support to the hypothesis that even small amounts of alcohol in utero have an effect on future cognitive outcomes’ (p. 7). In between, the authors often equate, implicitly or explicitly, fetal alcohol exposure with genetic variations, as in their statement of aim: ‘The purpose of this study was to determine whether exposure to moderate levels of alcohol during gestation influences child’s [*sic*] cognition’ (p. 4). This is an important leap of logic, since as the authors note, the size and direction of the effect of the relevant genetic mutations on the metabolisation of alcohol is in fact not known. No direct test is conducted (nor was possible) of whether these mutations were in fact associated with greater alcohol exposure in utero.

A related point arises about the manner in which the authors acknowledge that the effects of genotype appear small (as noted previously, Theme 6, Research Limitations). They write:

Whilst the effects of genotype appear modest … it is important to remember that these are effects for genotypes which are likely to result in very small differences in peak alcohol levels and alcohol exposure, and these subtle metabolic effects are among women drinking less than 1 unit of alcohol per day. Larger causal effects are anticipated for more substantial differences in fetal alcohol exposure levels, for example the differences existing between offspring of mothers with moderate alcohol consumption and mothers abstaining. (p. 7)

Rhetorically, the authors frame their acknowledgement of a small effect size as a claim that the effect size may be larger in reality. This implies that level of maternal alcohol intake would be a stronger determinant of fetal alcohol exposure than the genetic variations, and that the two factors would interact. However, no attempt is made to test for an interaction effect between amount consumed (among women who drink) and the genetic variations. Instead, the authors report breakdowns of the effects of genes among women who drink moderately and heavily but conclude that sample sizes are too small to be conclusive. Importantly, also, the authors’ speculation here links the amount of alcohol consumed by women to their children’s intelligence: this is a dose–response hypothesis that is not substantiated by their data.

To summarise our analysis thus far, the paper reports that there was a positive association between drinking in pregnancy and child IQ (Theme 1), confounding positive associations between both of those variables and SES (Theme 5), and limitations of the research (Theme 6). The paper also claims to resolve uncertainties surrounding previous research (Theme 7). Theme 4, the role of genes in moderating the effects of alcohol, is central to the paper. The paper does not explicitly indicate that drinking alcohol lowers child IQ (Theme 2), but there is ambiguity. It does not warn women not to drink (Theme 3). We now move to demonstrate how these themes and the interpretations of them discussed above developed in the risk story, and how other Themes 2 and especially 3 came to dominate the media coverage.

### Communication to the media: press releases and expert comments

#### The press release: ‘even moderate drinking in pregnancy can affect a child’s IQ’

The press release raised Theme 7 (addressing uncertainty) near the start, as follows:

Current advice to pregnant women about moderate alcohol consumption during pregnancy is contradictory, with some official guidelines recommending complete abstinence and others suggesting moderate use is safe. Previous studies have produced conflicting and inconsistent evidence on the effects of moderate alcohol intake on a child’s IQ. (University of Bristol, 2012)

As we have suggested already, the extent to which the research itself provides evidence that can be considered to address perceived inconsistency, and provide a clear response to conflict over the effect of moderate alcohol intake for child IQ, is debatable. However, there are three main ways in which the press release develops on Theme 7, through which claims are made that depart from the emphasis on limitations to providing certainty that is in the paper.

First, as Table 1 shows, no mention is made at all in the Press Release of Theme 1 (the finding that the children of mothers who drink have *higher* IQs than the children of those who do not). Rather, in so far as this matter is mentioned, it is presented as a problem that has characterised *other* research, but which has been addressed by this study, and so can be disregarded. The press release text thus compares the study with those that use ‘observational evidence’ and which have found ‘moderate drinking is beneficial compared to abstention’ but find this ‘because mothers who drink in moderation are typically well educated, have a good diet and are unlikely to smoke – all factors which are linked to higher IQ in the child’. In contrast, the research is presented as a step forward because it examined ‘alcohol intake in over 4000 women’ and, crucially, ‘used a novel technique known as Mendelian randomization’, which is ‘scientifically robust’ as it uses ‘genetic variants which modify exposure levels and which are not influenced by lifestyle and other factors’. In this way, the message is communicated that the research definitively addresses the limitations of other studies because it looks at DNA and ‘genetic variants’.

Second, commentary does not refute but rather appears to endorse Theme 2 (that evidence has now been found of a causal link between drinking at low-to-moderate levels and harm). As we have noted, this theme does not appear explicitly in the article, yet it does appear in the Press Release (which is titled: ‘Even moderate drinking in pregnancy can affect a child’s IQ’). Further, while the text states that, ‘variants in *alcohol metabolising genes*’ were related to ‘lower IQ at age eight’, it is stated immediately next that, ‘There was no effect evident among children whose mothers abstained during pregnancy, *strongly suggesting that it was the exposure to alcohol in the womb* that was leading to the difference in child IQ’ (our emphasis). Notably, comments included in the Press Release from authors of the paper are along these lines: for example, the results ‘suggest that even at levels of alcohol consumption which are normally considered to be harmless, we can detect differences in childhood IQ’ (Lewis), and, ‘This is evidence that even at these moderate levels, alcohol is influencing foetal brain development’ (Gray).

An unequivocal connection is made, third, between the research and advice to women (Theme 3). While this is not discussed in the article at all, the press release ends with Gray’s comment that although the study was ‘complex’, the message is ‘simple’, and this is

Even moderate amounts of alcohol during pregnancy can have an effect on future child intelligence. So women have good reason to choose to avoid alcohol when pregnant.

#### SMC commentary

The SMC produced two documents: a briefing note, ‘Alcohol consumption during pregnancy’ and a compilation of 11 short comments about the paper, ‘Expert reaction to new research into alcohol consumption during pregnancy’ (SMC, 2012a, 2012b). The first of these is most at odds with all other materials we examined, in that it contains no comment about the research addressing uncertainty (Theme 7). Rather, comment is limited to a succinct four-point ‘summary’ of the study and its findings, and comment on the study’s ‘strengths/limitations’. It highlights that the relationship identified in the study is between ‘alcohol metabolism and IQ in moderate drinkers’, not drinking and IQ (Theme 1). In regards to limitations (Theme 6), it is noted that ‘normal IQ range spans 20 points’ and that the ‘size of effect … −1.8 IQ points for moderate drinking in mothers who are poor alcohol metabolisers’ is ‘limited in size’. It is also stated that it is ‘large enough to have effects at a population level’ but that the article, ‘does not comment on whether it is a small effect on all children, or an occasional large effect on a few children’. A further limitation noted is that the paper does not compare between women who metabolise alcohol differently: ‘It is unclear what level of drinking in normal metabolisers corresponds to moderate drinking in poor alcohol metabolisers’, that is, the relation between alcohol consumption and child IQ for some women. Thus overall, the interpretation offered seems to be that that study is well designed (it is described as ‘a powerful study design’) but has limitations.

The points made by the 11 experts asked to comment by the SMC varied considerably. They raised addressing uncertainty (Theme 7):

There remains a lot of uncertainty about whether light or moderate drinking in pregnancy has an adverse effect on the fetus…. This paper … is scientifically important because it illustrates the use of a potentially powerful new way of answering this question. (Leon)

Description of findings (Theme 1) was commented on:

This study, like previous studies, actually found slightly lower IQ in children whose mothers drank no alcohol in pregnancy compared to those who drank moderately. However, the authors argued that this could be due to lower age and educational level among abstainers. (Bishop)

Interpretation of findings (Theme 2) was also emphasised in comments that the study, ‘does not provide an estimate of the actual effect of moderate drinking on children’s IQ’ (Speigelhalter), and ‘As the authors make clear, the statistical association they find is not the same as showing that drinking on pregnancy actually causes a reduction in childhood IQ’ (Leon). The theme of the moderating effect of genes and how to understand this (Theme 4) was emphasised by others (Collins, Tower).

Although it did not appear at all in the paper itself, Theme 3 (Warnings to women) featured strongly in some expert comment:

This research serves to confirm that drinking even a small amount of alcohol whilst pregnant can do harm to your own child … an estimated 6000 babies a year in the UK are born with brain damage, physical problems or learning disabilities as a result of heavy alcohol consumption by their mother. (Newell)

This comment ended with the words, ‘drink no alcohol at all’. Where other comment was made on this theme, noone suggested that the dangers of drinking in pregnancy are exaggerated. Three indicated, however, that the study provides no basis for making any new recommendations or changing policy (Bishop, Speigelhalter and Lang). Others emphasised anyway that ‘don’t drink’ is the best advice (O’Brien, Nicholls, Leon, Collins, Newell, Nutt). ‘Even though the IQ effects are small, if at all possible women should avoid ethanol in pregnancy as it’s a known toxin’, stated one expert (Nutt).

### Media coverage

Our analysis so far suggests that journalists did have some options in what to report about the research. An interpretation could have appeared, following some comment provided by the SMC and by some experts, that emphasised the children of women who do not drink have lower IQ than the children of women who do (Theme 1), possible evidence of modest moderating effect of genes (Theme 4) and research limitations (Theme 6). Alternatively, journalists had the option to take the story primarily from the Press Release and so highlight other themes, which as we now go on to discuss is what they did.

#### Representation of the findings (Themes 1 and 5)

As we have noted, the original article accurately stated that drinking during pregnancy was positively associated with children’s IQ (Theme 1), but the press release does not mention this finding. As shown in Table 1, of the 65 items of press coverage, 44 made no mention of this relation between drinking during pregnancy. Of the 21 items that did, two-thirds (14) got the direction of the findings wrong, stating that pregnant women who drank moderately during pregnancy had *less* intelligent children than those who abstained, while only a third (7) reported it accurately. A typical example of factually incorrect description of the findings is found in the *CBS News* report:

A new study found women who were moderate drinkers had children whose IQ was at least two points lower than women who didn’t drink.

Only five of the pieces mentioned the finding that SES was confounded with drinking during pregnancy and children’s IQ. Those who did, following Lewis et al. (2012), used this finding to explain the positive association between drinking in pregnancy and children’s IQ, or to highlight the uncertainties surrounding previous research. For example, *Bishop Blog* stated:

…in their study [Lewis et al.], the lowest IQs were obtained by children of mothers who did *not* drink at all during pregnancy. However, these mothers were also likely to be younger and less socially advantaged than mothers who drank, making it hard to disentangle causal influences (emphasis in the original).

#### Drinking harms children. Don’t drink! (Themes 2 and 3)

The most typical interpretation of the research was to ‘top and tail’ reporting with commentary suggesting that this study provides new evidence of the deleterious effects of drinking in pregnancy (Theme 2: 53 out of 65 items) and with warnings to women not to drink (Theme 3: 47 out of 65 items). The *Western Daily Press* stated, ‘Sip can damage unborn babies; New alcohol warning to mothers’, and the opening line of the report in *The Guardian* reads:

Research showing that children of women who drank as little as two glasses of wine a week during pregnancy had lower IQs has prompted calls for mothers-to-be to avoid alcohol.

Most articles made the warning the ‘take home message’. *Top News* told readers, ‘A new study has found that alcohol affects a child’s intelligence when the baby is in a mother’s womb. Therefore, expecting mothers should abstain from drinking, it has suggested.’ Warnings often included a quotation from a study author, and sometimes an additional expert, to make this point. Indeed, almost all articles contained a quotation of this sort, most frequently from one of the study authors. Gray (study co-author) was quoted, for example, in the following ways:

It is for individual women to decide whether or not to drink during pregnancy, we just want to provide the evidence. But I would recommend avoiding alcohol. Why take the risk? (*BBC News Online*)

[N]ow we know moderate alcohol does affect IQ. If you have a choice, why risk it? (*New Scientist*)

It was Theme 2, the attribution of causality for lowered IQ to alcohol *itself* that formed the dominant basis for these warnings. Despite the riders in the paper itself about this issue, almost all articles contained one of the following formulations, regarding drinking in pregnancy: Can affect a baby/child’s IQ; Harms a baby/child’s IQ; Lowers a baby/child’s IQ; Risks a baby/child’s IQ; and Damages a baby’s/child’s IQ. The message of a causal link was particularly clear in some reporting, especially in that using the words ‘risk’ and ‘harm’ as these headlines show:

Mothers-to-be risk child’s IQ with glass of wine a week. (*Irish Examiner*)

Moderate Drinking Will Damage Your Baby’s IQ. (*Direct2Mum*)

Just one glass of wine a week while pregnant ‘can harm a baby’s IQ.’ (*Daily Mail*)

Any wine and kid’s a plonker. (*The Sun*)

‘Two Wines’ harm unborn child. (*news.com.au*)

Even a tiny tipple in pregnancy can harm a child’s IQ. (*The Times*)

Alcohol in pregnancy can take six points off child’s IQ, claims study. (*The Daily Telegraph*)

Moderate drinking in pregnancy ‘harms IQ.’ (*BBC News Online*)

Moderate drinking will damage your baby’s IQ. (*Direct2Mum*)

One or two glasses of wine a week enough to harm unborn child. (*Herald Sun*)

There is a direct association between alcohol exposure in the womb and damage to a child’s IQ. (*Medical News Today*)

Drinking while pregnant ‘harms IQ.’ (*thedrinksbusiness.com*)

Drinking during pregnancy affects child’s intelligence. (*OnMedica*)

There was widespread use of the following comments from authors of the paper that appeared in the Press Release to emphasise this theme, neither of which mention either genes or the extent of IQ differences. The quote frequently used to link alcohol and harm was

Our results suggest that even at levels of alcohol consumption which are normally considered harmless we can detect differences in childhood IQ, which are dependent on the ability of the foetus to clear this alcohol. This is evidence that, even at these moderate levels, alcohol is influencing foetal brain development. (Lewis)

Women were warned against drinking through the idea of a causal link between drinking and lowered child IQ as follows:

Even moderate amounts of alcohol during pregnancy can have an effect on future child intelligence. So women have a good reason to choose to avoid alcohol when pregnant. (Gray)

Overall, the linking of alcohol itself to harm worked to minimise emphasis on uncertainty or limitations associated with the research. There is ‘good reason’ to ‘avoid alcohol’, readers were told, which is that evidence has been found that ‘alcohol can have an effect on child intelligence’. We now consider claims about certainty and uncertainty further and discuss two aspects to what was argued.

#### Genes, certainty, and uncertainty

The majority of media coverage (55 out of 65 items) referred to Theme 4, the moderating role of genes. *Channel 4 News* summarised this point as

Their results showed a lowering of IQ for those children whose genes are more susceptible to alcohol and whose mothers consumed between one and six units of alcohol per week.

In about half of the media coverage, a research focus on genes was represented in relation to Theme 7 (addressing uncertainty). It was posed as a definitive route to elimination of prior confusion and uncertainty; ‘factors’ that confound in other studies were ‘ruled out’ – it was claimed. For example, this was how *The Times* reported on the research:

Women who drink even a couple of glasses of wine a week during pregnancy are risking a two-point drop in their child’s IQ, a study suggests. The research is some of the strongest evidence yet in the furious debate that has seen mothers-to-be bombarded with contradictory advice on drinking. By using a novel technique of ‘genetic randomisation’, scientists were able to see through a statistical fog to conclude that most children have genes that make it risky for their mothers to drink even a small amount in pregnancy. The results should prompt women to think again about having any alcohol at all during pregnancy, experts said.

Similarly, *BBC News Online* and *Healthcare Today* emphasised how the research overcomes problems of contradiction, inconsistency, and confusion. (The point was expressed in a very similar way in these two cases, because the reporting drew directly on the press release, as did other such coverage raising this theme.):

Previous studies have produced inconsistent and confusing evidence on whether low to moderate levels of alcohol are harmful in pregnancy, largely because it is difficult to separate out other factors that may have an effect such as the mother’s age and education. But this research, published in the PLOS One journal, ruled that out by looking at changes in the genes that are not connected to social or lifestyle effects. (*BBC News Online*)

Previous studies have produced confusing evidence on the impact of moderate alcohol intake during pregnancy but these findings ruled out other factors that may have an effect, such as the mother’s age and education, by looking at changes in the genes that are not connected to social or lifestyle effects. (*Healthcare Today*)

This unequivocal account of research about ‘genes’ inevitably shifts the focus away from questions of SES and its effects for children and their development, to instead highlight the drinking habits of individual women and their relation to biology. It was this, in turn, that led to a second feature of what was claimed in reporting. This was that women, individually, have even more reason to be *uncertain* about the safety of drinking at low or moderate levels. The focus on genes made it possible to argue that a woman’s very biology means she can never know whether what she does is going to impair her child, and for this reason, she should not drink.

This emphasis on uncertainty at the level of the individual woman was captured in some reporting through the term ‘genetic lottery’, used by one expert who had commented for the SMC (Collins):

What do mums take from this? Unfortunately it’s a bit of a gene lottery. If your child has a particular gene profile, drinking any alcohol in pregnancy will have an effect on IQ – but, and it’s a big but – your child may not have one of those identified gene defects, and so the effect is negligible. (*Channel 4 News*)

While her comment emphasised a ‘big but’ and that it may be ‘the effect is negligible’, the emphasis of other reporting was, however, different. It was strongly and overtly in the direction of emphasising that uncertainty about your individual genes means, ‘don’t drink’. This was most clearly the case in reporting based on commentary more extensive than that in the press release, from the study co-author, Gray. For example, in reporting by *Fox News*, drinking ‘without harming babies’ is described as a theoretical possibility, but one about which there can never be certainty because a woman can never know what genes she possesses:

In theory, some women may be able to drink a glass of wine here or there during pregnancy without harming their babies, Gray said. But guidelines should still encourage all women during the nine months of pregnancy to skip that glass of beer with dinner, he added … ‘You could never in the real world analyze to find out who all had these genes and who didn’t. Pregnant women and women about to become pregnant don’t know which category they’re in. When you take that together it just strengthens the idea that it may be best for women to choose to avoid alcohol during pregnancy.’

There were very few examples of reporting that departed from the messages outlined above. There was a small amount of coverage in specialist medical and scientific publications. However, a strong emphasis on the ‘don’t drink’ message was ubiquitous in these cases; indeed, this message was pressed even more strongly in the specialist press than in the mainstream media.

Only five news articles included any discussion of Theme 6 (limitations of research). Almost all commentary on this issue was provided in the small number of comment pieces written about the paper. As we noted above, all of these comments were blog pieces, and they commonly offered an assessment of the research methodology and its limitations. One argued of the statistical work carried out, for example, that, ‘It is misleading, and not good practice, to just look at reported statistical significance, irrespective of how small the p value is and then infer causality’. This piece ended:

This work has all the hallmarks of data mining, no prescribed hypothesis, too many statistical tests, small insignificant differences found and inappropriate statistical methods employed. Therefore if you are asked for advice on this topic, which as a GP I often am, the current study adds little to current understanding. The advice, and the answer to our question is, drink moderately in pregnancy. (*Carl Heneghan’s Blog*)

There was only one piece out of the 65 analysed that made pregnant women the focus of the story, and this was a comment piece by the journalist Viv Groskop in the *Daily Mail*. ‘Another week, another lecture for the hapless pregnant woman’, it began. Linking this story to others about food, this episode of discussion about drinking and pregnancy was described as a ‘scare story’ serving ‘only to make pregnancy into a neurotic nightmare of guilt and self-loathing’. Risk, it was suggested, has not been discussed in balanced way, but in a way that ‘scares’ and leads to negative feelings in pregnant women.

## Discussion

As our analysis of the media coverage shows, three themes dominated reporting. These were democratised messages about harm to children caused by any drinking in pregnancy, warnings to all women about the need to abstain, and claims about research focused on genes providing new evidence. It was claimed that these messages were based on what had emerged from new research. While there were some alternative assessments offered of what ‘research shows’, these were few in number, and were mostly restricted to Blogs, not mainstream media reporting. On overall, finding was that this media representation of the research reported in ‘Fetal Alcohol Exposure and IQ at Age 8: Evidence from a Population-Based Birth-Cohort Study’ (Lewis et al., 2012) was out of line with what the article argued, most notably with its finding about the relation between lowered child IQ and drinking.

However, this disparity between the article and the reporting was not the whole picture. Although the dominant themes in media reporting did not accurately capture what was said in the article itself, we have shown that what happened to generate this outcome departs from some findings of other investigations. There is a ‘prevailing notion (not only amongst risk researchers) … that the media exaggerate some risks and ignore others … sacrificing objectivity for sensationalism’, suggest Wahlberg and Sjoberg (2000, p. 33). In this case though, it was not true overall that the account of risk that came to dominate reporting can be accurately described as simply ‘media misrepresentation’ or ‘sensationalism’. Rather, the reporting provided a *relatively* accurate account of what had been presented to journalists as the research findings, especially in the relative de-emphasis of the association between drinking in pregnancy and higher child IQ.

This was clear when we compared the press release to media reporting, and this concurs with Riesch and Spiegelhalter (2011) assessment of the role of press releases, and also that which emerged from a large study of press releases communicating about findings of published research about biomedical and health related topics (Sumner et al., 2014). As Sumner et al. noted:

Although it is common to blame media outlets and their journalists for news perceived as exaggerated, sensationalised, or alarmist, our principle findings were that most of the inflation detected in our study did not occur *de novo* in the media but was already present in the text of press releases produced by academics and their departments. (p. 4)

In the example considered here, our assessment goes further, however. What the media reported certainly did not ‘occur *de novo’* and was encouraged by the press release. More specifically, authors of the article emerged as playing an important role in communicating the messages about risk that dominated the coverage. The comments attributed to them in the press release emphasised themes that were absent from the article altogether (Theme 3, warnings to women) and omitted what can be considered important findings of the research (Theme 1, Description of findings, lower IQ in children of women who abstain from alcohol). Additionally, their comments highlighting an already ambiguous account of findings present in the article itself in regards to the attribution of causality for low IQ to drinking (Theme 2).

This may be considered consistent with a normal and uncontentious pattern of scientific story telling that is perhaps typical of many scientific papers: limitations and caveats are acknowledged, but in the context of a presentation style that tends to favour the authors’ preferred interpretation of their findings. In our case study, comments from the authors in the press release, however, took this further, made certain points less ambiguous, and encouraged a perception of a causal relation between drinking in pregnancy and lowered IQ in children. Notably, in the press release, the authors themselves also commented with warnings to women, although this was outside the scope of their research. The other source of comment provided to journalists to help them write about the research came from the SMC. The Centre’s own summary of the study accurately presented the findings and did not make any comment on warnings to women. While we did find some variations in the points made in experts’ comments, levels of engagement with the research findings varied considerably, however, and some explicitly suggested causality and warned women.

A further important feature of this risk story emerged from the analysis, which was the account given to the media (and so reported in the coverage) of the relation between Theme 7 (addressing uncertainty), Theme 5 (association between SES and IQ), and Theme 4 (the moderating role of genes). We noted above the fact that in the article itself, the authors did not present any findings of their own on Theme 5. They suggested in comments to the media, however, that their work nevertheless addressed uncertainty given by the association between SES and IQ and highlighted its distinctive and novel focus on genes. These were also themes emphasised in the press release.

What our analysis of the media coverage showed is how this presentation of genes, IQ, and SES led to a particular sort of argument about uncertainty. The message that research focused on genes gave new certainty about the dangers of drinking in pregnancy also comprised the claim that individual pregnant women should consequently be uncertain about the safety of drinking at all. It is in this regard that this risk story made a new contribution to the social organisation of risk. This comprised downplaying the importance of SES and other ‘social factors’ for child IQ, and emphasising instead the biology of the individual woman, the effects of which for her child she cannot be certain. Again, the media reporting of this claim about risk and uncertainty did not constitute simple exaggeration or misreporting but was in line with the messages of communication to the media.

## Conclusions

Riesch and Spiegelhalter concluded from their research that:

[T]he press release and the associated activities that the scientific institutions used to market their reports had a perceptible influence on how the issues were debated by the media, and allowed them to frame the stories around themes never really intended by the scientists. (2011, p. 49)

This assessment of the media as sensationalist in accounts of risk, and at odds with what scientists themselves intend, is one we particularly engage in this article. It is a long-standing theme in the sociological literature (Tulloch & Zinn, 2011), but in this paper, we suggest that a picture emerges with some differences.

As we have shown, the media reporting very clearly presented a democratised account of risk and warned women. It is true to say that this account differed considerably from the findings of the research, and that important factual inaccuracies were introduced by journalists. However, it is not at all clear that this difference arose from the media simply framing the story ‘around themes never really intended by the scientists’. Rather, the media coverage appears as a partly accurate reflection of the themes in the press release used to communicate to the media about the study, and these themes appear to arise, partly at least, from the initial interpretation of the research findings that can be found in the article itself. They were also reflected in some subsequent commentary provided by the authors of the original article.

We suggest in conclusion that our findings resonate with literature that has highlighted how experts themselves can discuss their research findings in such a way as to endorse existing precepts about risk. At no point did Lewis et al. (2012) factually misrepresent their findings. Further, while we have questioned some of their interpretations, researchers are entitled to draw debatable conclusions from their work and our results and analysis do not call into question the integrity of any researchers nor their right to advocate a particular interpretation of their work. Nonetheless, our work has demonstrated how researchers can shape risk narratives, by publicly favouring debatable interpretations and implications of their findings, and communicating through press releases that report some key findings but omit others. (In this case, this is true of authors of the research paper and is also true of some experts who offered comment then given to the media by the SMC.) We suggest that the result of this was that media coverage conveyed a strongly precautionary narrative, that is, one that suggests pregnant women need to become more risk aware and risk averse (Lowe & Lee, 2010). This message was stripped of the nuances and caveats of the original paper and bolstered by factual omissions and distortions that served to further close down any admission of scientific uncertainty.

Holland et al. (2011), also using a case study approach, have showed how this was similarly the case for reporting about obesity and Australia’s ‘Fat Bomb’. As they demonstrated, experts may frame their claims in relation to pre-existing accounts of risk that have come to dominate policy-making. What we have shown happened in this case also resonates particularly with Yeomans’ assessment of the larger recent history of evidence and policy for alcohol in general. He argues that an ‘expert marketplace’ operates that encourages the development of ‘strategies’ on the part of those seeking to influence policy-making. These have as one feature a competition to generate certainty where there is none. Yeomans concludes that as a result, research and what it is said to find can become, ‘connected to’ hype ‘or sensationalism on the part of experts and governments’ to the end of making uncertainty certain (2013, p. 73).

In sociological terms, this assessment points to the importance of the wider context that shapes claims that are made about certainty. Perhaps surprisingly, there has not been a great deal of research that considers media coverage of drinking in pregnancy. That which has been done, however, shows first a marked increase in the volume of media coverage this century; second that media coverage has become relatively less skeptical and more alarmist about claims that any drinking harms babies; and third that this media coverage is linked to campaigning activities of organisations promoting abstinence, changes to Department of Health policy, and also the publication of studies about research considering whether drinking impairs child development (Lowe, Lee, & Yardley, 2010). In other words, it suggests that there is an already existing, dominant context that encourages emphasis on the message ‘don’t drink in pregnancy’.

This means that those presently seeking to comment in public domains may arguably become less likely to draw attention to findings that contradict the consensus; in particular, they are unlikely to draw attention to the finding that children of women who abstain in pregnancy do relatively worse than the children of those who drink at low or moderate levels. However, serious attention should be paid to the effects of selective reporting of findings and absence of discussion of evidence that contradicts existing precepts. While positive evidence of harm may be what is considered both policy-relevant and newsworthy, the marginalisation of the question of generating unnecessary anxiety and diminished quality of life for women (matters raised by only one journalist in this case) is surely an important real-life casualty of risk stories about this issue.

## Appendix 1. Media coverage

**Table:**

News (UK and international print/online versions of print)
Guardian ‘IQ research prompts warning over drinking alcohol during pregnancy’
The Daily Telegraph (London) ‘Alcohol in pregnancy can take six points off child’s IQ, claims study’
The Independent ‘Even moderate drinking during pregnancy can affect child’s IQ’
The Times (London) ‘Warning tiny amount of alcohol during pregnancy can harm a child’s IQ’
The Sun (England) ‘Any wine and kid’s a plonker; MUMS WARNED’
Daily Mail (online) ‘Just one glass of wine a week while pregnant “can harm a baby’s IQ” ’
Huffington Post Women, UK ‘Mums-To-Be Advised To Avoid All Alcohol’
Huffington Post Parents ‘Light drinking while pregnant could lower baby’s IQ’
Metro ‘Drinking small glass of wine while pregnant “could reduce child’s IQ”’
Scotsman ‘Drinking one small glass of wine a week can lower child’s IQ, mothers-to-be warned’
Belfast Telegraph Online ‘Avoid alcohol, mums-to-be warned’
Irish Examiner ‘Mothers-to-be risk child’s IQ with glass of wine a week’
Irish Times ‘Alcohol In Pregnancy “Could damage IQ”’
Independent.ie ‘“Mums to be” warned just one glass of wine a week can reduce their child’s IQ’
Bristol Post ‘Mums warned of booze threat to babies’ IQ; Drinking while pregnant could harm children’
Western Mail ‘Pregnant women advised to avoid all alcohol’
Bath Chronicle ‘New alcohol warning to mothers … drop of wine can still harm your baby’
Western Daily Press ‘Sip can damage unborn babies; New alcohol warning to mothers Drop of wine can still harm baby, pregnant mums told’
Chicago Tribune ‘Even moderate drinking in pregnancy may affect child’s IQ’
Los Angeles Times ‘Moderate alcohol intake during pregnancy may not be OK after all’
Herald Sun ‘One or two glasses of wine a week enough to harm unborn child: study’
Daily Mail (online India) ‘Drinking even small amounts of alcohol while pregnant “can affect child’s IQ”’
India (Mail) Today ‘Alcohol consumed during pregnancy can affect child’s IQ: Study’
**News (UK and International Broadcast, online versions)**
BBC News online ‘Moderate drinking in pregnancy “harms IQ”’
BBC Newsbeat ‘Your view: Mums say they didn’t drink during pregnancy’
Channel 4 News ‘Drinking in pregnancy “harms baby’s IQ”’
Sky News ‘Alcohol In Pregnancy “Can Lower Child’s IQ”’
CBS News ‘Moderate drinking during pregnancy may lower child’s IQ’
Fox News ‘Light drinking while pregnant could lower baby’s IQ’
**News (blog/online only)**
RTT News ‘Avoid Alcohol When Pregnant And Here’s Why: Study’
Active quote ‘Drinking just one glass of wine a week during pregnancy could lower child IQ’
Direct 2 Mum ‘Moderate Drinking Will Damage Your Baby’s IQ’
Health Canal ‘Moderate drinking in pregnancy can affect child’s IQ’
Health Canal ‘RCOG statement on new research suggesting moderate alcohol consumption during pregnancy has an effect on children’s IQ’
Healthcare Today UK ‘Drinking in pregnancy can impact on IQ’
On Medica ‘Drinking during pregnancy affects child’s intelligence’
The Drinks Business ‘Drinking when pregnant harms IQ’
Thejournal.ie ‘Study shows even moderate drinking in pregnancy can affect child’s IQ’
News.com.au ‘Two wines’ harm unborn child’
Counsel & Heal ‘Drinking During Pregnancy Lowers Child’s IQ’
Insider Medicine ‘Moderate drinking during pregnancy affects child’s IQ’
Made for Mums ‘New study reinforces advice to avoid alcohol in pregnancy’
Medical Daily ‘Even Moderate Drinking During Pregnancy Lowers Child’s IQ’
Medical News Today ‘Even Moderate Drinking While Pregnant Can Hurt Child’s IQ’
Net Doctor ‘Moderate drinking in pregnancy “may affect IQ”’
Top News US ‘Expecting Mothers Should Avoid Drinking: Study’
US News ‘Moderate Drinking in Pregnancy Tied to Lower IQ in Child’
WebMD ‘Latest research on pregnancy and alcohol’
JournalWatch ‘Moderate Alcohol Consumption in Pregnancy Linked to Lower Childhood IQ, Genotypying Study Suggests’
IOL Lifestyle ‘Drinking while pregnant can affect baby’s IQ’
Reuters ‘Even moderate drinking in pregnancy may affect child’s IQ’
Yahoo News ‘Light Drinking While Pregnant Could Lower Baby’s IQ’
Live Science ‘Light Drinking While Pregnant Could Lower Baby’s IQ’
News.com.au ‘Kids’ IQs affected by small alcohol intake’
**NGO/Government body/specialist press**
Wellcome Trust ‘Moderate drinking in pregnancy can affect a child’s IQ’^1^
New Scientist ‘Moderate drink during pregnancy can lower baby’s IQ’
Nursing Times ‘Warning over moderate drinking during pregnancy’
NCADD ‘Moderate Levels of Drinking in Pregnancy Linked With Lower IQ in Children’
RCOG statement ‘RCOG statement on new research suggesting moderate alcohol consumption during pregnancy has an effect on children’s IQ’
**Comment pieces**
BishopBlog ‘Moderate drinking in pregnancy: toxic or benign?’
Skeptical Scalpel ‘Skeptical Scalpel: Moderate maternal alcohol use lowers children’s IQ (not)’
Carl Heneghan ‘Should I drink moderately during pregnancy?’
Understanding Uncertainty (David Spiegelhalter) ‘Alcohol in pregnancy and IQ of children’
NHS Choices ‘Weekly glass of wine in pregnancy “harms kids’ IQ”’^2^
MailOnline ‘What’s worse for a baby … a sip of Pinot Grigio or a guilt-ridden mum?’^3^

^1^Is identical to the press release. ^2^Also falls under the category of ‘NGO/Government body/specialist press’. ^3^Also falls under the category of ‘News (UK and international print /online versions of print)’. RCOG: Royal College of Obstetricians and Gynaecologists; NCADD: National Council on Alcohol & Drug Dependence.

## Appendix 2. Themes and sub-themes present in documents

**Table:**

	Media coverage *(65 articles)*	Science Media Centre *(12 pieces)*^1^	Press release	**Lewis et al.’s (2012)****article**
Theme code and description	*Number of articles where theme was present*		*Theme present? 1 = yes, 0 = no*	
**1. Description of findings**				
Says_IQ_higher_if_drink: States that empirically, children of mothers who drink have higher IQs	7	1	0	1
Says_IQ_lower_if_drink: States that empirically, children of mothers who drink have lower IQs	14	0	0	0
**2. Interpretation of findings**				
Says_alc_harmful: States that there is a causal, deleterious link between drinking and IQ (separately from whether or how it describes the actual findings)	56	3	1	1
Says_causal_link_not_proven: States that the study does not demonstrate a causal link	5	3	0	1
If_alc_harmful_definite: *If it says alcohol is harmfu*l, does it state that the findings demonstrate a causal link without qualification? That is, there is an effect (not could be)	53	1	1	0
If_alc_harmful_maybe: *If it says alcohol is harmfu*l, does it state that the findings suggest there *could* be a causal link? (Elsewhere, in the article, there might have been a definite causal statement, which can also be coded)	37	2	1	1
**3. Warnings to women**				
Warning: Contains a warning or advice not to drink	47	6	1	0
If_warning_precautionary: *If so*, is this warning merely precautionary? (i.e., findings not clear/risk not known, therefore exercise ‘the precautionary principle’ and avoid the unknown, possible risk)	17	4	0	0
If_warning_findings: *If so*, is warning allegedly based on findings? (i.e., findings show that is safest for women not to drink)	40	1	1	0
**4. Role of genes in moderating effects of alcohol**				
Gene_effect_moderated: Mentions the role of genes	55	4	1	1
**5. Association between IQ, drinking in pregnancy and socio-economic status**				
Mentions-SES: Mentions the role of SES	5	1	0	1
If_SES_poshmumsdrink: *If the story mentions SES*, does it state that high SES mums drink more?	5	1	0	1
If_SES_confound: *If the story mentions SES*, does it state that SES is confounded with drinking? (i.e., it is positively correlated not only with drinking but also with children’s IQ)	1	1	0	1
If_SES_vague_wrong: *If the story mentions SES*, does it do so in a vague or incorrect way? (e.g., mentioning SES but not saying in what direction it works, or even implying that low SES is associated with drinking)	0	0	0	0
**6. Methodological limitations**				
Methodological_critique: Contains methodological critique of the article	8	4	0	1
Effect size small?: Mentions that the effect sizes observed are small	16	2	0	0
Effect size large?: Mentions that the effect sizes observed are large	0	0	0	0
**7. Addressing uncertainty in other research**				
Positioning_trumps previous research/eliminates confusion and uncertainty?: Claims that the present findings eliminate or resolve previous contradiction and uncertainty in research	30	1	1	1
Positioning_present findings should be interpreted/weighed with caution given other results: Claims that the present findings should be taken with a pinch of salt or not weighed too heavily given that other studies have shown no (or beneficial) effects	5	1	0	0
Claim that method rules out lifestyle and socio-economic effects: Claims that the method eliminates influence of other factors … similar to trumping previous research but doesn’t require story to refer to other research, just takes current study alone	29	1	1	1

Note: ^1^This includes the SMC summary and 11 expert commentaries.

## References

[CIT0001] Alati R., Macleod J., Hickman M., Sayal K., May M., Smith G. D., & Lawlor D. A. (2008). Intrauterine exposure to alcohol and tobacco use and childhood IQ: Findings from a parental offspring comparison within the Avon longitudinal study of parents and children. *Pediatric Research*, 64, 659–666. 18670372 10.1203/PDR.0b013e318187cc31

[CIT0002] Armstrong E. M. (1998). Diagnosing moral disorder: The discovery and evolution of fetal alcohol syndrome. *Social Science and Medicine*, 47(12), 2025–2042. 10075244 10.1016/s0277-9536(98)00308-6

[CIT0003] Armstrong E. M. (2003). *Conceiving risk, bearing responsibility, fetal alcohol syndrome and the diagnosis of moral disorder*. Baltimore, MD: John Hopkins University Press.

[CIT0004] Armstrong E. M., & Abel E. L. (2000). Fetal alcohol syndrome: The origins of a moral panic. *Alcohol and Alcoholism*, 35(3), 276–282. 10869248 10.1093/alcalc/35.3.276

[CIT0005] Bell K., McNaughton D., & Salmon A. (2009). Medicine, morality and mothering: Public health discourses on foetal alcohol exposure, smoking around children, and childhood overnutrition. *Critical Public Health*, 19(2), 155–170.

[CIT0006] Department of Health (2016). *Alcohol guidelines review – Report from the guidelines development group to the UK chief medical officers*. London: Department of Health Retrieved from https://www.gov.uk/government/consultations/health-risks-from-alcohol-new-guidelines

[CIT0007] Golden J. (1999). “An argument that goes back to the womb”: The demedicalization of fetal alcohol syndrome, 1973–1992. *Journal of Social History*, 33, 269–298.

[CIT0008] Golden J. (2005). *Message in a bottle, the making of fetal alcohol syndrome*. Cambridge, MA: Harvard University Press.

[CIT0009] Hammer R., & Inglin S. (2014). ‘I don’t think it’s risky, but….’: Pregnant women’s risk perceptions of maternal drinking and smoking. *Health, Risk & Society*, 16(1), 22–35. doi:10.1080/13698575.2013.863851

[CIT0010] Henderson J., Kesmodel U., & Gray R. (2007). Systematic review of the fetal effects of prenatal binge-drinking. *Journal of Epidemiology & Community Health*, 61, 1069–1073. doi:10.1136/jech.2006.054213 18000129 PMC2465662

[CIT0011] Holland K., Blood R. W., Thomas S. I., Lewis S., Komesaroff P. A., & Castle D. J. (2011). ‘Our girth is plain to see’: An analysis of newspaper coverage of Australia’s future ‘Fat Bomb’. *Health, Risk & Society*, 13(1), 31–46. doi:10.1080/13698575.2010.540648

[CIT0012] Holland K., McCallum K., & Walton A. (2016). I’m not clear on what the risk is’: Women’s reflexive negotiations of uncertainty about alcohol during pregnancy. *Health, Risk & Society*, 18(1–2), 38–58. doi:10.1080/13698575.2016.1166186

[CIT0013] Humphriss R., Hall A., May M., Zuccolo L., & Macleod J. (2013). Prenatal alcohol exposure and childhood balance ability: Findings from a UK birth cohort study. *BMJ Open*, 3(6), e002718. doi:10.1136/bmjopen-2013-002718 PMC3686236 23794556

[CIT0014] Kelly Y. J., Sacker A., Gray R., Kelly J., Wolke D., Head J., & Quigley M. A. (2012). Light drinking during pregnancy: Still no increased risk for socioemotional difficulties or cognitive deficits at 5 years of age? *Journal of Epidemiology and Community Health*, 66, 41–48. doi:10.1136/jech.2009.103002 20924051

[CIT0015] Lee E. (2014). Policing pregnancy: The pregnant woman who drinks In Lee E., Bristow J., Faircloth C., & Macvarish J. (Eds.), *Parenting culture studies* (pp. 129–146). Basingstoke: Palgrave Macmillan.

[CIT0016] Leppo A., Hecksher D., & Tryggvesson K. (2014). ‘Why take chances?’ Advice on alcohol intake to pregnant and non-pregnant women in four Nordic countries. *Health, Risk & Society*, 16(6), 512–529. doi:10.1080/13698575.2014.957659

[CIT0017] Lewis S., Zuccolo L., Davey Smith G., Macleod J., Rodriguez S., Draper E. S., … Thornton-Wells T. A. (2012). Fetal alcohol exposure and IQ at age 8: Evidence from a population-based birth cohort study. *PLoS One*, 7(11), e49407. doi:10.1371/journal.pone.0049407 23166662 PMC3498109

[CIT0018] Lowe P., & Lee E. (2010). Advocating alcohol abstinence to pregnant women: Some observations about British policy. *Health, Risk & Society*, 12(4), 301–311. doi:10.1080/13698571003789690

[CIT0019] Lowe P., Lee E., & Yardley L. (2010). Under the influence? The construction of foetal alcohol syndrome in UK newspapers. *Sociological Research Online*, 15(4), 2. doi:10.5153/sro.2225

[CIT0020] Lupton D. (2012). ‘Precious cargo’: Foetal subjects, risk and reproductive citizenship. *Critical Public Health*, 22(3), 329–340. doi:10.1080/09581596.2012.657612

[CIT0021] Lupton D. (2014). *The social worlds of the unborn*. Basingstoke: Palgrave Macmillan.

[CIT0022] Murphy A. O., Sutton R. M., Douglas K. M., & McClellan L. M. (2011). Ambivalent sexism and the “do”s and “don’t”s of pregnancy: Examining attitudes toward proscriptions and the women who flout them. *Personality and Individual Differences*, 51, 812–816. doi:10.1016/j.paid.2011.06.031

[CIT0023] O’Callaghan F. V., O’Callaghan M., Najman J. M., Williams G. M., & Bor W. (2007). Prenatal alcohol exposure and attention, learning and intellectual ability at 14 years: A prospective longitudinal study. *Early Human Development*, 83, 115–123. doi:10.1016/j.earlhumdev.2006.05.011 16842939

[CIT0024] Potter D. A. (2012). Drawing the line at drinking for two: Governmentality, biopolitics, and risk in state legislation on fetal alcohol spectrum disorders. *Critical Perspectives on Addiction*, 14, 129–153.

[CIT0025] Science Media Centre (2012a). *Alcohol consumption during pregnancy* . Retrieved from http://www.sciencemediacentre.org/expert-reaction-to-new-research-into-alcohol-consumption-during-pregnancy-2/

[CIT0026] Riesch H., & Spiegelhalter D. J. (2011). ‘Careless pork costs lives’: Risk stories from science to press release to media. *Health, Risk & Society*, 13(1), 47–64. doi:10.1080/13698575.2010.540645

[CIT0027] Ruhl L. (1999). Liberal governance and prenatal care: Risk and regulation in pregnancy. *Economy and Society*, 28(1), 95–117. doi:10.1080/03085149900000026

[CIT0028] Science Media Centre (2012b). *Expert reaction to new research into alcohol consumption during pregnancy* . Retrieved from http://www.sciencemediacentre.org/expert-reaction-to-new-research-into-alcohol-consumption-during-pregnancy/

[CIT0029] Science Media Centre (homepage) (2016). Retrieved 314, 2016, from http://www.sciencemediacentre.org/

[CIT0030] Smyth C. (2012, 11 15). Warning tiny amount of alcohol during pregnancy can harm child’s IQ. *The Times*. Retrieved from http://www.thetimes.co.uk/tto/health/news/article3600232.ece

[CIT0031] Sumner P., Vivian-Griffiths S., Boivin J., Williams A., Venetis C. A., Davies A., … Boy F. (2014). The association between exaggeration in health related science news and academic press releases: Retrospective observational study. *British Medical Journal*, 349, g7015. doi:10.1136/bmj.g7015 25498121 PMC4262123

[CIT0032] Sutton R. M., Douglas K. M., & McClellan L. (2011). Benevolent sexism, perceived health risks, and the inclination to restrict pregnant women’s freedoms. *Sex Roles*, 65, 596–605. doi:10.1007/s11199-010-9869-0

[CIT0033] Tulloch J. C., & Zinn J. (2011). Risk, health and the media. *Health, Risk & Society*, 13(1), 1–16. doi:10.1080/13698575.2010.543123

[CIT0034] University of Bristol (2012). *Even moderate drinking in pregnancy can affect a child’s IQ* . Retrieved from http://bristol.ac.uk/news/2012/8936.html

[CIT0035] Wahlberg A. A. F., & Sjoberg L. (2000). Risk perception and the media. *Journal of Risk Research*, 3(1), 31–50. doi:10.1080/136698700376699

[CIT0036] Yeomans H. (2013). Blurred visions: Experts, evidence and the promotion of moderate drinking. *The Sociological Review*, 61(S2), 58–78. doi:10.1111/sore.2013.61.issue-s2

